# Full-Genome Sequence of a Novel Varicella-Zoster Virus Clade Isolated in Mexico

**DOI:** 10.1128/genomeA.00752-15

**Published:** 2015-07-09

**Authors:** Fabiola Garcés-Ayala, Araceli Rodríguez-Castillo, Joanna María Ortiz-Alcántara, Elizabeth Gonzalez-Durán, José Miguel Segura-Candelas, Sandra Ivette Pérez-Agüeros, Noé Escobar-Escamilla, Alfonso Méndez-Tenorio, José Alberto Diaz-Quiñonez, José Ernesto Ramirez-González

**Affiliations:** a Departamento de Biología Molecular y Validación de Técnicas, Instituto de Diagnóstico y Referencia Epidemiológicos (InDRE), Secretaría de Salud, México City, México; b Facultad de Medicina, UNAM, México City, México; c Laboratorio de Bioinformática y Biotecnología Genómica, Escuela Nacional de Ciencias Biológicas (ENCB), Instituto Politécnico Nacional, México City, México

## Abstract

Varicella-zoster virus (VZV) is a member of the *Herpesviridae* family, which causes varicella (chicken pox) and herpes zoster (shingles) in humans. Here, we report the complete genome sequence of varicella-zoster virus, isolated from a vesicular fluid sample, revealing the circulation of VZV clade VIII in Mexico.

## GENOME ANNOUNCEMENT

The varicella-zoster virus (VZV) (also known as human herpesvirus 3) virion consists of a nucleocapsid surrounding a core that contains the genome comprised of 125 kb of linear double-stranded DNA containing approximately 71 open reading frames (ORFs) (1). The viral genome is similar to that of other alphaherpesviruses, consisting of two unique coding regions, unique long (UL) and unique short (US), each flanked by inverted repeats; short repeats termed terminal repeat long (TRL) and internal repeat long (IRL) border the unique long region, while larger repeats termed terminal repeat short (TRS) and internal repeat short (IRS) border the unique short region (2).

As a function of geographic distribution, VZV has been separated into the five major clades 1 to 5 confirmed by full-genome sequencing (3–5). In addition, four novel clades VI to IX have been proposed that still to be confirmed by further complete sequences. These provisional clades have been rarely reported to date (3, 4). In Mexico, clade 1 (genotype E1), clade 3 (genotype E2), and clade 5 (genotype M1) have been reported based on the sequence analysis of a fragment of ORF 22 (6).

Total nucleic acid extraction of Var160 strain was carried out directly from a vesicular fluid sample and the genetic material amplified using an Illustra Genomi Phi v2 DNA amplification kit. The preparation of a genomic library was performed with a Nextera DNA sample preparation kit followed by clonal amplification and sequencing in the MiSeq instrument. As a result, 2,094,929 reads with an average length of 150 bp were obtained, which corresponded to 316 Mbp. The reads were assembled using GS Reference Mapper version 2.0, with the sequence of the Dumas strain as the mapping reference (NC_001348). Forty contigs between 90 and 85,000 bp were generated, and an average coverage of 2000× was obtained. The annotation of the sequence was performed, resulting in the identification of 72 genes and coding sequences (CDS).

Preliminary phylogenetic analysis was performed by using MEGA5.0 with the neighbor-joining method, and the results demonstrated that the Var160 strain belongs to clade VIII, revealing the presence of this novel clade in Mexico. This is the first report of the complete genome sequence of a VZV isolated in Mexico and a detailed sequence and phylogenetic analyses of this genome will be published elsewhere.

### Nucleotide sequence accession number.

The varicella-zoster virus isolate Var160 genome sequence has been deposited in GenBank under the accession no. KC112914.
